# Burden and Predictions of Untreated Caries in China, 1990–2039: A Systematic Analysis for the Global Burden of Disease Study 2019

**DOI:** 10.1002/cre2.70211

**Published:** 2025-09-08

**Authors:** Xiaolei Zhang, Chan‐Na Zhao, Hu Zheng, Chunhui Zhao, Yuanyin Wang, Hai‐Feng Pan, Wuli Li

**Affiliations:** ^1^ College & Hospital of Stomatology Anhui Medical University, Key Lab. of Oral Diseases Research of Anhui Province Hefei Anhui China; ^2^ Department of Epidemiology and Biostatistics, School of Public Health Anhui Medical University Hefei Anhui China

**Keywords:** deciduous teeth, dental caries, disease burden, permanent teeth

## Abstract

**Objective:**

This study aims to analyze the burden of untreated caries in deciduous and permanent teeth in China from 1990 to 2019 and projects its future trends through 2039.

**Materials and Methods:**

Data on the burden of caries in primary and permanent teeth in China between 1990 and 2019 were extracted from the Global Burden of Disease 2019 study to assess the current burden of untreated caries. The average annual percentage change (AAPC) was estimated using a joinpoint regression model to evaluate temporal trends in the burden of untreated caries. A Bayesian age–period–cohort model was applied to project the burden of untreated caries between 2019 and 2039.

**Results:**

From 1990 to 2019, the age‐standardized incidence rate (ASIR) of untreated caries in deciduous teeth in China increased by 6.8%, with an AAPC of 0.22 (95% confidence interval [95% CI]: 0.15–0.22, *p* < 0.05). The ASIR of caries in permanent teeth decreased by 1.3%, with an AAPC of −0.05 (95% CI: −0.04 to −0.05, *p* < 0.05). The highest incidence of caries in primary teeth was observed in the 0–4 age group, whereas the highest ASIR of caries in permanent teeth was observed in the 20–24 age group. No significant sex differences were found in the incidence rates of caries in either dentition. Projections from 2020 to 2039 indicate an upward trend in the ASIR of caries in both deciduous and permanent teeth.

**Conclusions:**

Over the past 30 years, the burden of dental caries in China has increased significantly. Projections indicate that the ASIR of dental caries will continue to increase. Therefore, appropriate prevention and control strategies are required to reduce the burden of caries in the Chinese population.

## 1 Introduction

The 2019 Global Oral Health Status Report (Sun et al. 2024; Wen et al. 2022; Organization 2022) shows that untreated caries in permanent teeth is the most prevalent oral disease, followed by untreated caries in deciduous teeth. These conditions affect approximately 2 billion adults and 530 million children, respectively. If left untreated, dental caries can lead to complications such as toothache, tooth loss, and odontogenic infections and may also increase the risk of systemic diseases, including infective endocarditis and coronary artery disease (Sen et al. 2024; Nomura et al. 2020). Dental caries pose a threat to individual health, resulting in a significant economic burden (D'Souza et al. 2022). In 2019, the global cost of direct treatment for dental diseases was estimated at approximately 387 billion US dollars (Jevdjevic and Listl 2025). To promote global oral health, the World Health Organization (WHO) released the Global Strategy and Action Plan on Oral Health 2023–2030, aiming to achieve a relative reduction in the overall prevalence of major oral diseases and conditions throughout life by 2030 (Organization 2024). Dental caries is listed by WHO as the third most common chronic noncommunicable disease worldwide (Organization 2022; Peres et al. 2019). The goal of joint global efforts is to reduce the prevalence of dental caries.

Over the past 30 years, the Chinese government has prioritized the prevention and management of caries. Since 2008, it has implemented a nationwide comprehensive intervention program targeting the oral health of children (National Health Commission of the People's Republic of China 2011), particularly in low‐income areas. This program includes preventive measures such as the use of pit and fissure sealants and topical fluoride, which have contributed to reducing the burden of dental caries among children. However, the results of the Fourth National Oral Health Survey in 2017 (The Central People's Government of the People's Republic of China 2017) indicated that in 2015, the prevalence of dental caries in primary teeth among 5‐year‐old children nationwide was 70.9%, an increase of 5.8% compared to that of a decade earlier. Among 12‐year‐old children, the prevalence of dental caries in permanent teeth was 34.5%, an increase of 7.8% compared to that of a decade earlier. In older adults aged 65–74 years, the prevalence of dental caries in permanent teeth was as high as 98.0%. These findings reflect the limited effectiveness of current dental caries prevention and management strategies in China. Therefore, closely examining the burden and trends of dental caries and developing practical and effective policies based on this analysis are essential.

The Global Burden of Disease (GBD) study uses a systematic and standardized approach to quantify the health and societal losses caused by diseases, disabilities, injuries, and premature death. Its analytical findings provide essential evidence to support health policy development strategies, optimal allocation of medical resources, and determination of priority areas in medical research (Kassebaum et al. 2017). Based on the GBD 2019 database, a joinpoint regression model was used in this study to analyze temporal trends in the burden of untreated dental caries in China from 1990 to 2019. A Bayesian age–period–cohort model was constructed to forecast the burden of untreated dental caries in China from 2019 to 2039.

## 2 Materials and Methods

### 2.1 Data Sources

The Global Burden of Disease (GBD) 2019 assessed 369 diseases and injuries in 204 countries and territories (Vos et al. 2020), accomplished through extensive data provision, review, and analysis. Every estimate was presented with 95% uncertainty intervals (95% UIs), indicating statistical significance. 95% UIs were determined based on the 2.5th‐ and 97.5th‐ordered percentiles of 1000 draws of the uncertainty distribution. This study estimated each epidemiological quantity of interest‐incidence, prevalence, and years lived with disability (YLDs). The age‐standardized incidence rate (ASIR), age‐standardized prevalence rate (ASPR), and age‐standardized YLDs rate (ASYR) were calculated for dental caries in deciduous and permanent teeth. Data on caries of deciduous teeth and caries of permanent teeth burden in China from 1990 to 2019 were obtained from the Global Health Data Exchange GBD Results Tool (https://vizhub.healthdata.org/gbd-results/). China's population censuses, conducted every 10 years (in 1990, 2000, and 2010), were the main source of demographic data by age and gender. In addition, data from the national population sample surveys conducted every 5 years were integrated, and a standardized methodology was used to estimate the population data of the other years. The annual and single‐year age estimates of net migration and population in China were generated using a Bayesian hierarchical cohort component model. The standard population structure used in age standardization was generated using the estimated age structures of the population for selected national‐level populations with at least 5 million in each group.

### 2.2 Definitions

This study included dental caries in deciduous teeth of the population aged 0–14 years and permanent teeth of the population aged ≥ 5 years. In accordance with the WHO (Vos et al. 2020), the Global Burden of Disease (GBD) study defines untreated caries as unfilled teeth with unmistakable cavity at the dentin or cementum level. Teeth that meet this definition are included in the criteria. In the GBD database, untreated caries corresponded to the ICD‐9 code 521.0 and the ICD‐10 codes K02.3‐K02.9.

### 2.3 Statistical Analysis

The GBD 2019 study (Vos et al. 2020) used a variety of disease and injury outcome models for estimation, including the Cause of Death Ensemble model (CODEm), spatiotemporal Gaussian process regression (ST‐GPR), and Bayesian meta‐regression tool DisMod‐MR, to estimate prevalence, incidence, mortality, years of life lost (YLLs), years lived with disability (YLDs), and disability‐adjusted life years (DALYs) across different causes, ages, sexes, years, and regions.

To assess the magnitude and direction of temporal trends in the rates for untreated caries, we calculated the average annual per cent change and corresponding 95% confidence intervals by joinpoint regression. The joinpoint regression model is a collection of linear statistical models that were used to evaluate the trends in disease burdens attributable to caries of deciduous teeth and permanent teeth from 1990 to 2019. Joinpoint (version 4.9.1.0; National Cancer Institute, Rockville, MD, USA) was used to create this model (Joinpoint Trend Analysis Software, Version 4.9.1.0—April 2022; Surveillance research program, Division of Cancer Control & Population Sciences, National Cancer Institute. Available from: https://surveillance.cancer.gov/joinpoint/). This model's calculating approach is to estimate the changing rule of illness rates using the least square method, avoiding the non‐objectivity of typical trend analyses based on linear trends. Calculation of the square sum of the residual error between the estimated and actual values yields the turning point of the shifting trend. Joinpoint regression analysis could identify the points with remarkable changes in trend (i.e., joinpoints), divide the overall trend into multiple subsegments based on these observed joinpoints, and further assess the epidemiological trend of each subsegment by calculating the annual percentage change (APC) and 95% confidence interval (CI). The average APC (AAPC), which serves as a summary measure of the trend over a prespecified fixed interval (1990–1999, 2000–2009, 2010–2019, and 1990–2019), was also calculated as a weighted average of APC by the span width of segmented interval (Clegg et al. 2009). We investigated if the fluctuation trend in different parts was statistically significant by comparing the AAPC or APC to 0. A statistically significant *p* value was less than 0.05.

Data on incidence, prevalence, and YLDs were described by year. We collected and reported the crude, age‐standardized estimates, and corresponding 95% uncertainty intervals (95% UIs) based on GBD 2019. Uncertainty is estimated using Monte Carlo simulations. The burden of untreated dental caries was stratified by age (5‐year age intervals) and sex. To ensure the representativeness of the sample, the GBD study typically uses a stratified random sampling method. The specific steps are as follows: (1) Stratification: The population is divided into different strata based on factors such as geographic region, age, sex, and socioeconomic status. (2) Random Sampling: Samples are randomly selected within each stratum to ensure that the probability of each individual being selected is known and equal. (3) Weight Adjustment: The sample data are weighted according to the sampling probabilities and the distribution of the target population to reflect the situation of the entire population. Only YLDs were used in this study, because it is uncommon that deaths are directly attributed to dental caries. YLDs were calculated by multiplying the frequency (prevalence), severity (disability weight), and duration of the condition. Finally, age‐standardized rates were calculated by sex and age to measure the direction of temporal trends of diseases and a Bayesian age–period–cohort (BAPC) analysis in *R* (Jürgens et al. 2014). Based on the GBD data from 1990 to 2019, we used the BAPC package in R software (Du et al. 2020) to predict the burden of dental caries from 2019 to 2039. BAPC uses integrated nested Laplace approximations (INLA) for full Bayesian inference. BAPC generates age‐specific and age‐standardized projected rates. When interest lies in the predictive distribution, Poisson noise is automatically added. All data analyses were conducted using R software (version 4.2.1; Bell Laboratories; Murray Hill, NJ, USA) and the Joinpoint Regression Program (version 4.9.0.0).

This study adheres to the Strengthening the Reporting of Observational studies in Epidemiology (STROBE) and Guideline for Accurate and Transparent Health Estimates Reporting (GATHER) guidelines to ensure transparent and reproducible reporting. In accordance with the STROBE requirements, we clearly described the data sources, inclusion/exclusion criteria, and statistical methods (Von Elm et al. 2014). Meanwhile, following the GATHER guidelines, we detailed the input data used in the GBD analysis, the modeling methods, and the assessment of uncertainty. This study was reported in a standardized manner according to the “Guidelines for Reporting Oral Epidemiological Studies,” specifying diagnostic criteria and sampling methods, and adjusting for potential biases (such as inter‐examiner differences and nonresponse bias) (Stevens et al. 2016). Additionally, in accordance with the GROSEBE guidelines, we have included relevant descriptions in the text (Bernabé et al. 2025).

## 3 Results

### 3.1 Burden of Untreated Caries in Deciduous Teeth in China

#### 3.1.1 Burden of Untreated Caries in Deciduous Teeth From 1990 to 2019

Table 1 shows that the number of incident cases of untreated dental caries in primary teeth in China declined by 25.7% from 184,782,763.4 (95% uncertainty interval [UI]: 128,630,735.9–244,055,709.7) in 1990 to 137,318,634.2 (95% UI: 98,321,622.4–176,631,393.7) in 2019. However, during the same period, the crude incidence rate increased by 6.8%, from 57,224.2 per 100,000 population (95% UI: 57,197.6–57,250.9) to 61,090.8 per 100,000 (95% UI: 61,057.9–61,123.6). The number of prevalent cases also declined, falling by 28.7% from 94,263,760.8 (95% UI: 75,712,510.7–112,734,407.7) in 1990 to 67,172,111.9 (95% UI: 54,028,765.4–79,658,270.3) in 2019. Despite this reduction, the crude prevalence rate increased slightly by 2.4% from 29,192.0 per 100,000 population (95% UI: 18,195.8–38,855.6) in 1990 to 29,883.8 per 100,000 (95% UI: 18,862.2–39,647.7). Similarly, the total number of YLDs attributable to untreated caries in primary teeth decreased by 28.6% from 36,246.0 (95% UI: 15,714.0–76,183.7) to 25,868.4 (95% UI: 11,287.7–54,496.0). Nevertheless, the crude YLD rate experienced a marginal increase of 2.7%, from 11.2 to 11.5 per 100,000 population (95% UI: 4.9–23.6 and 5.0–24.2, respectively). The age‐standardized YLD rate (ASYR) increased by 1.8% from 11.3 (95% UI: 4.5–23.4) to 11.5 (95% UI: 4.6–23.9).

**Table 1:** Cases and their age‐standardized rate of incidence, prevalence, and YLDs and their percentage change for untreated deciduous caries in China from 1990 to 2019.

	Incidence			Prevalence			YLDs		
	1990	2019	Change	1990	2019	Change	1990	2019	Change
	(95% UI)	(95% UI)	(%)	(95% UI)	(95% UI)	(%)	(95%UI)	(95%UI)	(%)
	(per 100,000)	(per 100,000)		(per 100,000)	(per 100,000)		(per100000)	(per100000)	
**Cases**									
Male	96,510,998.6	73,908,529.6	−23.4%	49,407,059.5	36,137,491.8	−26.9%	19,000.8	10,605.7	−44.2%
	(67,624,816.6–127,328,894.8)	(52,563,347.8–95,339,955.6)		(39,808,536.1–58,932,494.7)	(29,028,553.1–42,846,927.5)		(8,274.0–39,860.1)	(4,103.5–23,790.0)	
Female	88,271,764.82	63,410,104.7	−28.2%	44,856,701.3	31,034,620.2	−30.8%	17,245.3	9,599.8	−44.3%
	(60,922,597.62–116,719,556.43)	(45,362,000.0–81,445,446.1)		(36,122,665.5–53,659,165.4)	(24,997,908.3–36,808,695.7)		(7,470.2–35,970.1)	(3,836.8–21,005.6)	
Both	184,782,763.4	137,318,634.2	−25.7%	94,263,760.9	67,172,111.9	−28.8%	36,246.0	25,868.4	−28.6%
	(128,630,735.9–244,055,709.7)	(98,321,622.4–176,631,393.7)		(75,712,510.9–112,734,407.7)	(54,028,765.4–79,658,270.3)		(15,714.0–76,183.7)	(11,287.7–54,496.0)	
Rate									
All ages	57,224.2	61090.8	6.8%	29,192.0	29883.8	2.4%	11.2	11.5	2.7%
	(30477.7–86262.3)	(34809.4–89512.8)		(18195.8–38855.6)	(18862.2–39647.7)		(4.5–23.3)	(4.6–23.9)	
Age‐standardized	57984.7	61917.3	6.8%	29291.4 (18333.1–39022.1)	29815.9 (18910.6–39614.0)	1.8％	11.3	11.5	1.8%
	(30880.1–87357.5)	(35294.2–90693.4)					(4.5–23.4)	(4.6–23.9)	

*Note:* 95% UI: 95% uncertainty interval.

**Figure 1:**
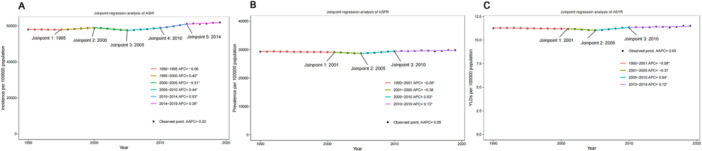
Joinpoint analysis of the trends in age‐standardized rates of incidence (A), prevalence (B), and YLDs (C) for caries in deciduous teeth in China from 1990 to 2019. AAPC < 0 indicates a downward trend, AAPC > 0 indicates an upward trend; * indicates *p* < 0.05; and AAPC, Average Annual Percentage Change. YLDs, Years lived with disability.

From 1990 to 2019, the age‐standardized incidence rate (ASIR) of untreated dental caries in primary teeth increased by 6.8%, from 57,984.7 per 100,000 population (95% UI: 30,880.1–87,357.5) to 61,917.3 per 100,000 (95% UI: 35,294.2–90,693.4) (Table 1). The AAPC was 0.22 (95% CI: 0.15–0.28; *p* < 0.05) (Figure 1). Joinpoint regression identified five inflection points in 1995, 2000, 2005, 2010, and 2014. The ASIR declined from 1990 to 1995, with an APC of −0.06% and from 2000 to 2005 (APC: −0.15; *p* < 0.05). However, from 2005 to 2010, from 2010 to 2014, and from 2014 to 2019, the ASIR showed an upward trend, with APCs of 0.40 (*p* < 0.05), 0.44 (*p* < 0.05), and 0.93 (*p* < 0.05), respectively. The age‐standardized prevalence rate (ASPR) showed a modest increase of 1.8% from 29,291.4 per 100,000 population (95% UI: 18,333.1–39,022.1) in 1990 to 29,815.9 (95% UI: 18,910.6–39,614.0) in 2019. The AAPC was 0.05% (95% CI: −0.03 to 0.12; *p* > 0.05). Three joinpoints were identified in 2001, 2005, and 2010. The ASPR declined from 1990 to 2001 (APC: −0.08; *p* < 0.05) and from 2001 to 2005 (APC: −0.38), followed by increases from 2005 to 2010 (APC: 0.53; *p* < 0.05) and from 2010 to 2019 (APC: 0.13; *p* < 0.05). Similarly, the ASYR increased from 11.3 per 100,000 population (95% UI: 4.5–23.4) in 1990 to 11.5 in 2019 (95% UI: 4.6–23.9), a 1.8% increase. The AAPC was 0.05 (95% CI: −0.31 to 0.22; *p* > 0.05). Three joinpoints were identified in 2001, 2005, and 2010. The ASYR declined from 1990 to 2001 (APC: −0.08; *p* < 0.05) and from 2001 to 2005 (APC: −0.37; *p* < 0.05), then increased from 2005 to 2010 (APC: 0.54%; *p* < 0.05) and from 2010 to 2019 (APC: 0.12, *p* > 0.05), respectively.

#### 3.1.2 Age‐ and Sex‐Stratified Analysis of the Burden of Dental Caries in Deciduous Teeth in 2019

**Figure 2:**
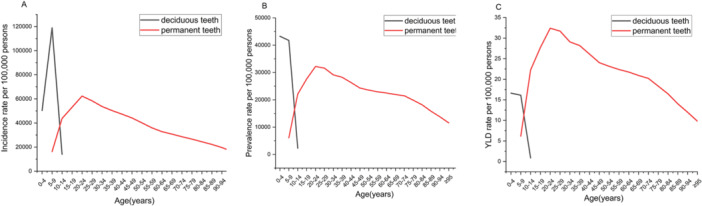
Incidence rate (A), prevalence rate (B), and YLD rate (C) of dental caries among different age groups in 2019. The incidence (A), prevalence (B), and YLD (C) rate of caries in deciduous teeth are the highest among children aged 5‐9 years; the incidence (A), prevalence (B), and YLD (C) rate of dental caries in permanent teeth are the highest among individuals aged 20–24 years. YLDs, Years lived with disability.

The ASIR of dental caries in primary teeth in 2019 first increased before decreasing with age, reaching a peak of 118,959.2 (95% UI: 118,909.1–119,009.3) in the 5–9 age group (Figure 2). The ASPR was the highest in the 0–4‐year age group at 43,265.9 per 100,000 population (95% UI: 43,237.4–43,294.4). The ASYR showed no significant changes across different age groups.

**Figure 3:**
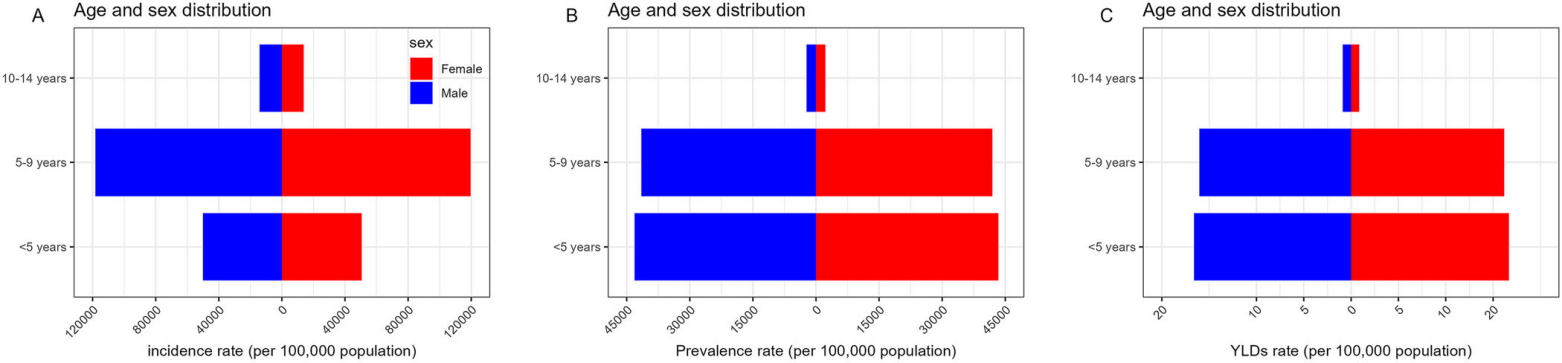
Age‐standardized rates of incidence (A), prevalence (B), and years lived with disability (C) of untreated caries in deciduous teeth in different gender and age groups in China, 2019. There are no significant gender differences in the rates of incidence (A), prevalence (B), and YLDs (C) across all age groups. YLDs, Years lived with disability.

The sex‐stratified analysis indicates that in 2019, no significant differences occurred between the sexes in the ASIR, prevalence rate, and YLDs of dental caries in primary teeth across all age groups (Figure 3).

### 3.2 Burden of Untreated Caries in Permanent Teeth in China

#### 3.2.1 Burden of Untreated Caries in Permanent Teeth in China From 1990 to 2019

Table 2 shows that the number of incident cases of untreated dental caries in permanent teeth in China increased by 17.4%, from 494,498,379.8 (95% UI: 443,960,842.5–544,539,848.3) in 1990 to 580,601,994.3 (95% UI: 528,464,825.9–637,158,148.8) in 2019. Despite this increase, the crude incidence rate decreased slightly by 2.3%, from 41,776.2 per 100,000 people (95% UI: 37,506.7–46,003.9) to 40,819.9 per 100,000 (95% UI: 37,154.3–44,796.1). Over the same period, the number of prevalent cases increased by 15.8%, from 285,005,145.4 (95% UI: 244,054,791.2–329,972,149.8) to 330,136,487.4 (95% UI: 285,836,779.5–381,128,922.0). However, the crude prevalence rate declined from 24,077.9 per 100,000 population (95% UI: 20,618.3–27,876.8) to 23,210.6 per 100,000 (95% UI: 20,096.1–26,795.7). The number of YLDs increased by 15.07%, from 283,459.8 (95% UI: 127,311.8–547,139.6) to 326,163.1 (95% UI: 143,273.5–633,994.8). The crude YLD rate decreased by 4.3%, from 23.95 (95% UI: 10.76–46.22) to 22.93 (95% UI: 10.07–44.57). The ASYR increased from 11.3 (95% UI: 4.5–23.4) to 11.5 (95% UI: 4.6–23.8).

**Table 2:** Age‐standardized rates of incidence, prevalence, and YLDs of untreated permanent caries in China from 1990 to 2019.

	Incidence rate			Prevalence rate			YLDs rate		
	1990	2019	Change	1990	2019	Change	1990	2019	Change
	(95％ UI)	(95％ UI)	(％)	(95％UI)	(95％ UI)	(％)	(95％ UI)	(95％ UI)	(％)
	(per 100,000)	(per 100,000)		(per 100,000)	(per 100,000)		(per 100,000)	(per 100,000)	
Cases									
Male	255,749,617.3	292,545,421.7	14.4%	143,545,258.4	163,495,373.5	13.9%	143,313.2	162,218.9	13.2%
	(230,344,475.1–281,508,618.3)	(266,367,889.1–320,576,620.0)		(123,332,008.5–166,164,725.9)	(141,403,611.0–189,555,626.2)		(63,576.1–276,263.9)	(71,479.8–315,553.2)	
Female	238,748,762.5	288,056,573.1	20.65%	141,459,887.0	166,641,113.9	17.8%	140,146.6	163,944.2	17.0%
	(213,186,135.6–263,528,969.7)	(262,073,378.3–316,573,510.1)		(120,610,951.6–163,337,868.2)	(143,651,047.5–191,504,166.7)		(62,929.7–269,938.1)	(72,037.3–318,409.3)	
Both	494,498,379.8	580,601,994.3	17.4%	285,005,145.4	330,136,487.4 (285,836,779.5–381,128,922.0)	15.8%	283,459.8 (127,311.8–547,139.6)	326,163.1	15.1%
	(443,960,842.5–544,539,848.3)	(528,464,825.9–637,158,148.8)		(244,054,791.2–329,972,149.8)				(143,273.5–633,994.8)	
Rate									
All ages	46288.8	43300.7	−6.5%	26678.7	24621.3	−7.7%	26.5	24.3	−8.3%
	(35695.5–56009.1)	(34280.1–51705.5)		(18890.7–37455.6)	(17577.8–34619.4)		(10.8–54.6)	(9.9–50.2)	
Age‐standardized	39,705.5	39,190.2	−1.3%	25717.1	24000.9	−6.7%	25.5	23.8	−6.7%
	(35,614.9–43,920.1)	(35,282.0–43,103.8)		(18037.2–36369.5)	(16996.3–33676.1)		(10.4–52.7)	(9.6–49.2)	

*Note:* 95% UI: 95% uncertainty interval.

**Figure 4:**
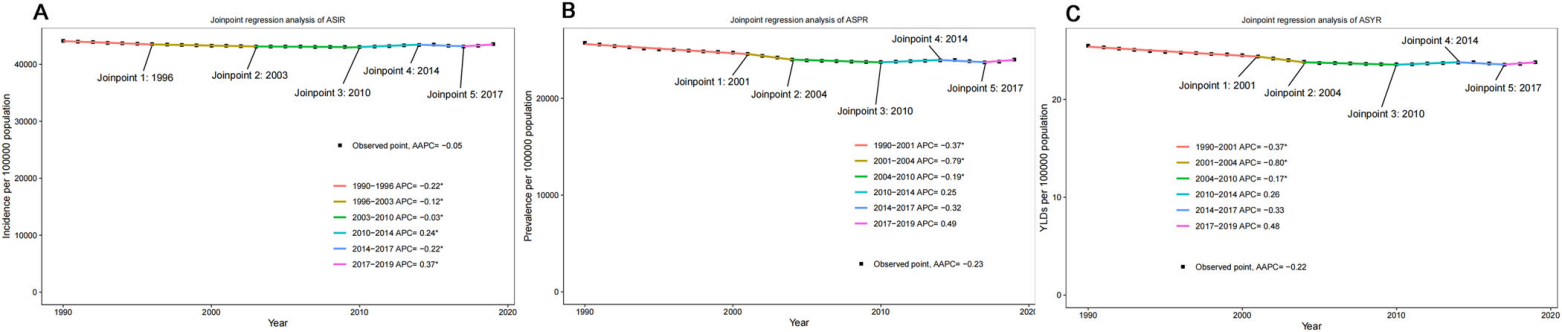
Joinpoint analysis of the trends in age‐standardized rates of incidence (A), prevalence (B), and YLDs (C) for caries in permanent teeth in China from 1990 to 2019. AAPC < 0 indicates a downward trend and AAPC > 0 indicates an upward trend; *indicates *p* < 0.05. AAPC, Average Annual Percentage Change.

From 1990 to 2019, the ASIR of untreated dental caries in permanent teeth declined by 1.3%, from 39,705.5 (95% UI: 35,614.9–43,920.1) to 39,190.2 per 100,000 population (95% UI: 35,282.0–43,103.8) (Table 2). The AAPC was −0.05 (95% confidence interval [CI]: −0.04 to −0.05; *p* < 0.05) (Figure 4). Joinpoints were observed in 1996, 2003, 2010, 2014, and 2017. An upward trend was observed between 2010 and 2014 (APC = 0.24; *p* < 0.05) and from 2017 to 2019 (APC = 0.37; *p* < 0.05). Downward trends were observed from 1990 to 1995, from 1996 to 2003, from 2003 to 2010, and from 2014 to 2017, with APCs of −0.22 (*p* < 0.05), −0.12 (*p* < 0.05), −0.03 (*p* < 0.05), and −0.22 (*p* < 0.05), respectively. From 1990 to 2019, the ASPR decreased by 6.7%, from 25,717.1 (95% UI: 18,037.2–36,369.5) to 24,000.9 (95% UI: 16,996.3–33,676.1), with AAPC of −0.23 (95% CI: −0.32 to −0.14; *p* < 0.05). Four joinpoints were identified in 2001, 2004, 2010, and 2017. From 2010 to 2014 and from 2017 to 2019, it showed an upward trend, with APCs of 0.25 and 0.49, respectively. However, from 1990 to 2001, from 2001 to 2004, from 2004 to 2010, and from 2014 to 2017, a downward trend was observed, with APCs of −0.37 (*p* < 0.05), −0.79 (*p* < 0.05), −0.19 (*p* < 0.05), and −0.32, respectively. The ASYR declined from 25.5 (95% UI: 10.4–52.7) to 23.8 (95% UI: 9.6–49.2), with an AAPC of −0.22 (95% CI: −0.31 to −0.13; *p* > 0.05). From 1990 to 2001, from 2001 to 2004, from 2004 to 2010, and from 2014 to 2017, the ASYR declined, with APCs of −0.37 (*p* < 0.05), −0.80 (*p* < 0.05), −0.17 (*p* < 0.05), and −0.33 (*p* < 0.05), respectively. Conversely, it increased from 2010 to 2014 and from 2017 to 2019, with APCs of 0.26 and 0.48, respectively.

#### 3.2.2 Age‐ and Sex‐Stratified Analysis of the Burden of Dental Caries in Permanent Teeth in 2019

Figure 2 shows that in the age‐stratified analysis, the age‐standardized incidence rate (ASIR) and prevalence of untreated dental caries in permanent teeth in 2019 first increased, before decreasing with age. The highest values occurred in the 20–24 age group, with an incidence rate of 62,301.5 per 100,000 population (95% UI: 53,022.2–69,104.6) and a prevalence of 32,195.0 per 100,000 population (95% UI: 25,702.8–41,915.4). The YLD rate showed no significant changes across different age groups.

**Figure 5:**
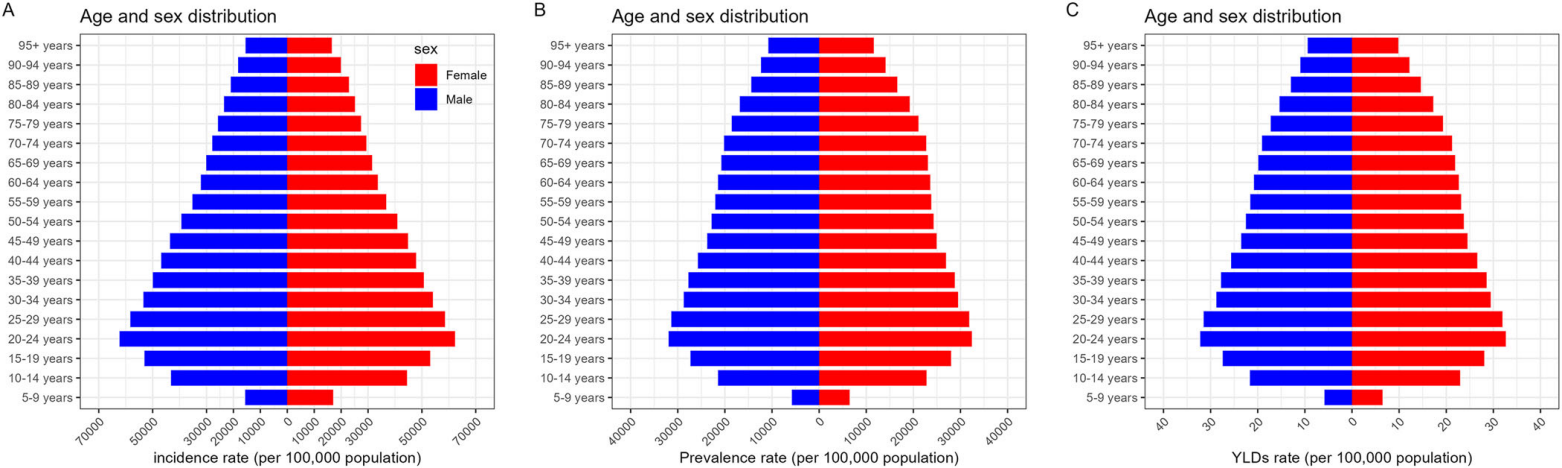
Age‐standardized rates of incidence (A), prevalence (B), and YLDs (C) of untreated caries in permanent teeth in different sex and age groups in China, 2019. There are no significant sex differences in the rates of incidence (A), prevalence (B), and YLDs (C) across all age groups. YLDs, Years lived with disability.

Figure 5 shows that in the sex‐stratified analysis, no significant differences were observed between males and females in the age‐standardized incidence, prevalence, and YLD rate of dental caries in permanent teeth in 2019.

### 3.3 Forecast of the Burden of Untreated Dental Caries in Deciduous and Permanent Teeth From 2019 to 2039

**Figure 6:**
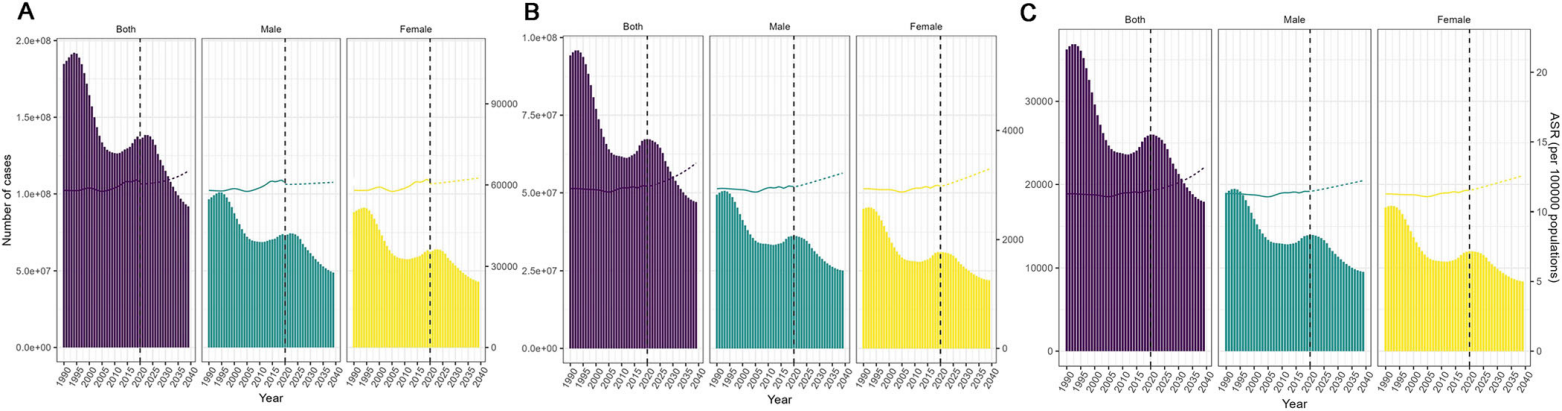
Trends in age‐standardized incidence (A), prevalence (B), and YLDs (C) rates of caries in deciduous teeth in China from 1990 to 2019, and projected trends from 2019 to 2039. The bar chart represents the number of cases and the line chart represents the trend of change in ASR. ASR, age‐standardized rates.

Figure 6 illustrates that, between 2019 and 2039, the crude incidence rate of dental caries in primary teeth among children in China is projected to increase from 61,090.8 per 100,000 population (95% uncertainty interval [UI]: 61,057.9–61,123.6) to 62,293.67 per 100,000 population (95% UI: 20,651.6–103,935.8). The age‐standardized incidence rate is expected to increase from 61,917.3 per 100,000 population (95% UI: 61,884.2–61,950.4) to 65,112.5 per 100,000 population (95% UI: 21,332.8–108,889.2), indicating an upward trend.

**Figure 7:**
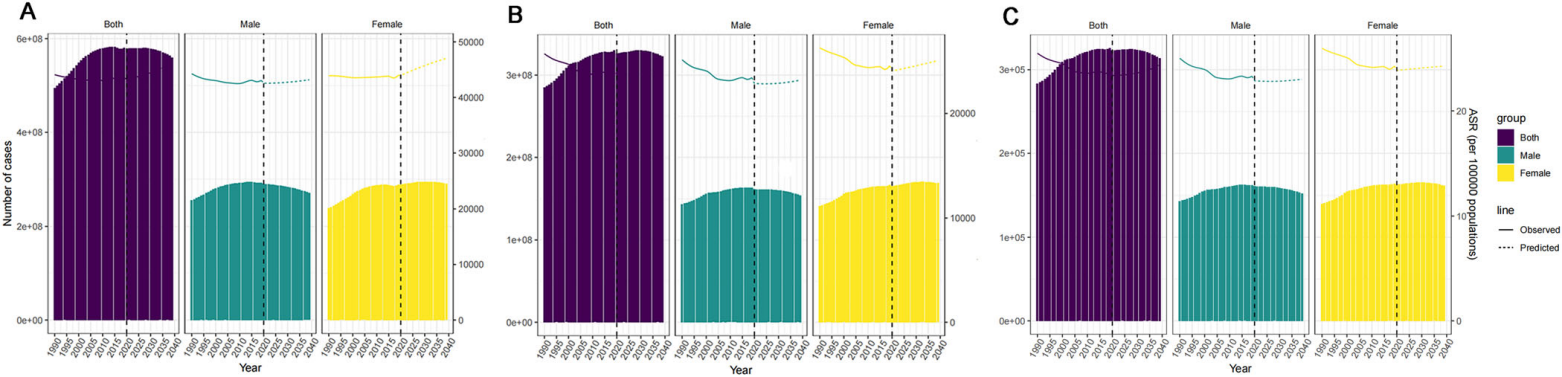
Trends in age‐standardized incidence (A), prevalence (B), and YLDs (C) rates of caries in permanent teeth in China from 1990 to 2019, and projected trends from 2019 to 2039. The bar chart represents the number of cases and the line chart represents the trend of change in ASR. ASR, age‐standardized rates.

Figure 7 shows that, from 2019 to 2039, the crude incidence rate of dental caries in permanent teeth among adults in China is projected to increase from 43,300.7 per 100,000 population (95% UI: 43,273.3–43,328.1) to 43,422.3 per 100,000 population (95% UI: 24,179.4–62,665.3). The age‐standardized incidence rate is expected to increase from 43,516.1 per 100,000 population (95% UI: 43,488.0–43,544.1) to 46,229.9 per 100,000 population (95% UI: 25,295.7–67,164.1), indicating an upward trend.

## 4 Discussion

Dental caries have a high incidence and cause extensive harm, making them a serious public health challenge in China and globally. To prevent and control dental caries, many regions globally have implemented various policies, such as water fluoridation, oral health education, and sugar reduction initiatives (World Health Organization 2015; Botchey et al. 2015; Pucca et al. 2015). However, research findings indicate that the global burden of dental caries has remained largely unchanged over the past three decades (Sun et al. 2024; M. Zhang, Zhang, et al. 2020). Studies show that the incidence, prevalence, and YLDs associated with dental caries continue to increase, highlighting failures in oral disease prevention and control (Benzian and Beltrán‐Aguilar 2025). In May 2025, the World Health Assembly called for a global strategy to address oral diseases and to integrate oral health into the noncommunicable diseases and universal health coverage agendas. These actions reflect a growing recognition of the severity of oral diseases and a commitment to reducing the prevalence of dental caries. Global Burden of Disease (GBD) studies have provided a basis for developing global dental caries prevention strategies. This study aimed to analyze trends in the burden of dental caries on primary and permanent teeth in China. The results will provide a reference for the formulation of dental caries prevention strategies in China.

This study showed that from 1990 to 2019, the number of incidents, prevalent cases, and YLDs owing to untreated dental caries in primary teeth in China decreased, whereas the crude incidence, crude prevalence, and crude YLD rates increased. This may be associated with a slowdown in population growth in China and a rising proportion of children (National Bureau of Statistics 2021). Over the same period, the crude incidence, prevalence, and YLD, alongside the age‐standardized incidence rates of untreated dental caries in primary teeth, showed statistically significant upward trends. This aligns with the results of the Fourth National Oral Health Epidemiological Survey in China (The Central People's Government of the People's Republic of China 2017) and the global trend reported in 2021 for primary tooth diseases (Li et al. 2025). Possible explanations for these trends include excessive sugar consumption and poor oral hygiene habits, which are important risk factors in the pathogenesis of dental caries. Consumption of sugary drinks and sweets among Chinese residents is steadily increasing (Sheiham and James 2014), whereas adoption of effective oral hygiene behaviors, such as brushing teeth twice daily, remains low (Kumar et al. 2016). The Fourth National Oral Health Survey reports that less than 25% of 5‐year‐old children brush their teeth twice daily (The Central People's Government of the People's Republic of China 2017). Targeted prevention efforts addressing excessive sugar intake and poor oral hygiene could improve effectiveness in controlling dental caries. Additionally, limited coverage of preventive healthcare may also contribute to the increased incidence of dental caries in primary teeth among children. Compared with urban areas, children in rural regions experience a higher incidence of dental caries (K. Zhang, Li, et al. 2020; Wen et al. 2025) owing to limited awareness of oral health and reduced access to medical resources. Efforts to reduce sugar consumption and increase the use of fluoride for caries prevention should be prioritized in rural areas to reduce the incidence of dental caries in primary teeth in China. From 1990 to 2019, the age‐standardized incidence rate of dental caries in primary teeth showed an upward trend, whereas the age‐standardized prevalence rate declined. These opposing trends indicate improvements in the treatment rate of dental caries in primary teeth. This may reflect improvements in economic development and educational levels in China. Awareness regarding caries in the primary teeth has changed, and more caregivers now recognize its effect and actively seek timely treatment (Anil and Anand 2017).

The incidence, prevalence, and number of YLDs owing to untreated dental caries in permanent teeth are increasing, whereas the crude incidence, prevalence, and YLD rates are declining—a trend consistent with the global burden of permanent tooth caries reported in 2021 (Bernabe et al. 2025). This seemingly paradoxical phenomenon may be directly caused by demographic changes (National Bureau of Statistics 2021). In 2019, the total population of China was 1.23 times larger than that in 1990; however, the absolute number of children aged 0–14 decreased by 240 million, whereas the population aged ≥ 15 increased correspondingly (The National Bureau of Statistics 2021), contributing to an increase in the number of cases of incidence, prevalence, and YLDs of dental caries in permanent teeth. Age‐standardized incidence, prevalence, and YLD rates showed a statistically significant downward trend. These suggest that economic development and improved education levels have contributed to greater awareness of oral health, and preventive efforts are beginning to yield results. In addition, the expansion and improved distribution of healthcare services have enabled more people to receive timely and effective treatment, leading to an increase in the treatment rate of dental caries.

The age‐stratified analysis revealed that in 2019, children aged 0–4 had the highest prevalence and years lived with disability (YLD) rate from dental caries among those aged 0–14. These findings are consistent with the global burden of dental caries in primary teeth reported in the GBD 2021 (Li et al. 2025). One possible explanation is that younger children are generally less cooperative during dental treatment, often making general anesthesia the only viable option. However, this technique is complex, difficult to implement, and currently not widely feasible in clinical practice (Knapp et al. 2021). Consequently, dental caries in children are not effectively treated, and their prevalence remains high. Many Chinese scholars have investigated the etiological factors contributing to dental caries in primary teeth (Xu et al. 2025; Lin et al. 2023), and their findings indicate that the development of dental caries in children is closely associated with sugar consumption and inadequate oral hygiene. The age‐standardized incidence rate (ASIR) of dental caries in permanent teeth in China initially increases with age, peaks in the 20–24 age group, and then gradually declines. Similarly, global data from 2021 indicate that individuals aged 20–24 experienced the highest burden of dental caries in permanent teeth (Bernabe et al. 2025). In contrast, the burdens among the elderly population (aged ≥ 65) are significantly lower than those observed in the younger population. This finding does not imply that the oral health status of older adults is optimistic. Tooth loss may obscure the history of dental caries, potentially resulting in an underestimation of its current prevalence among adults. According to the Fourth National Oral Health Epidemiological Survey in China, the treatment rate of dental caries in the elderly population is low, with only a small proportion receiving restorative (filling) treatment. Owing to limitations such as socioeconomic status, health awareness, physical condition, and other related factors, elderly individuals typically have a lower level of awareness regarding dental caries and often seek medical attention only when the condition leads to toothaches.

The results of the sex‐stratified analysis showed that between 1990 and 2019, no significant difference was observed in the burden of dental caries in primary teeth between males and females in China. Similarly, studies using the GBD 2017 (GBD 2017 Oral Disorders Collaborators 2020) and GBD 2021 (Bernabe et al. 2025) databases showed no significant sex‐based differences in the global burden of dental caries. However, epidemiological evidence suggests that the prevalence of dental caries is higher in females than in males. One possible reason is that female teeth tend to erupt earlier than those of males, resulting in longer exposure to the oral environment and an increased risk of bacterial erosion. Additionally, differences in the supragingival microbiota have been observed between males and females with severe early childhood caries, indicating that sex may influence the composition of plaque microbiota (Shaffer et al. 2015). The causes are related to hormones, saliva, and other biological factors (De Jesus et al. 2020). Studies show that increasing estrogen levels are associated with an increase in the incidence of dental caries, whereas increases in androgen levels do not have an effect (Lukacs 2011). During periods of hormonal fluctuation—such as puberty, menstruation, and pregnancy—the biochemical composition and secretion rate of saliva change significantly (Martinez‐Mier and Zandona 2013). Furthermore, females tend to have smaller salivary glands and a lower average saliva secretion rate than those of males, which further increases their risk of developing dental caries.

The forecast results from this study show that the incidence, prevalence, and YLD rates of dental caries in China will continue to increase from 2019 to 2039. By 2039, the incidence of dental caries in primary and permanent teeth is projected to reach 65,112.5 and 46,229.9 per 100,000 population, respectively. The prevalence is expected to reach 34,012.3 and 24,841.9 per 100,000, whereas the YLD rates are expected to be 13.1 and 24.4 per 100,000, respectively. These findings indicate that the future burden of dental caries in China remains high, highlighting the urgent need for effective preventive and intervention strategies.

This study has some limitations. First, the definition of untreated dental caries used in the GBD 2019 Study follows the World Health Organization criteria, which only considers teeth with visible cavities having caries. However, early‐stage (incipient) caries without cavitation should also be included in caries management, as they can be reversed through remineralization therapy, potentially reducing treatment costs. However, the GBD lacks data on early‐stage caries. Second, the data used in this study were sourced from the GBD 2019 Study, some of which are based on estimates rather than direct measurements, potentially introducing inaccuracies. Third, the GBD 2019 may have incomplete data for certain years, and specific information on dental caries in individual Chinese provinces may be missing. This could affect the accuracy of the overall analysis and limit the assessment of regional disease burden. Fourth, in some regions or for certain diseases, only limited data may be available, resulting in wide 95% uncertainty intervals and increased uncertainty. Additionally, these intervals are sensitive to extreme values or outliers, which can further widen the intervals and influence the interpretation and reliability of the study results.

In summary, this study showed trends in the burden of dental caries in China from 1990 to 2019 and predicted the burden from 2020 to 2039. The results show that over the past three decades, the burden of dental caries in China has remained largely unchanged, and this trend is expected to continue without large‐scale, effective interventions. Future work should focus on the following areas: First, implementing targeted preventive measures based on identified risk factors, such as expanding the application of fluoride and reducing sugar consumption; second, prioritizing young children and adults by strengthening disease surveillance and organizing regular community‐based screenings; third, exploring the establishment of an oral health insurance system to reduce the financial burden of treatment and encourage more public awareness and proactive prevention of dental caries; and finally, optimizing the allocation of medical resources and strengthening the medical security service system to reduce the overall burden of dental caries in the Chinese population.

## Permission to Reproduce Material From Other Sources

The data used in this study were obtained from the Global Burden of Disease (GBD) database, which is publicly available and maintained by the Institute for Health Metrics and Evaluation (IHME). The data can be accessed through the Global Health Data Exchange (GHDx) website.

## Author Contributions

**Xiaolei Zhang, ChanNa Zhao:** writing original draft, data collection, and formal analysis. **Zheng Hu, Chunhui Zhao:** data curation, formal analysis, and validation. **Yuanyin Wang, Haifeng Pan** and **Wuli Li:** conceptualization, methodology, supervision, review, and editing. The final version has been reviewed, edited, and approved by all authors.

## Ethics Statement

The ethical review determined that this study did not require approval because it used publicly available data.

## Consent

Informed consent was waived for this study.

## Conflicts of Interest

The authors declare no conflicts of interest.

## Data Availability

The data sets [GENERATED/ANALYZED] for this study can be found in the [Global Health Data Exchange GBD Results Tool] [https://vizhub.healthdata.org/gbd-results/].
